# Tibial torus and toddler's fractures misdiagnosed as transient synovitis: a case series

**DOI:** 10.1186/1752-1947-5-305

**Published:** 2011-07-13

**Authors:** Aksel Seyahi, Serkan Uludag, Burak Altıntaş, Mehmet Demirhan

**Affiliations:** 1 American Hospital, Department of Orthopaedics and Traumatology, Istanbul, Turkey; 2 Istanbul University, Istanbul Faculty of Medicine; Department of Orthopedics and Traumatology, Istanbul, Turkey

## Abstract

**Introduction:**

The high incidence of transient synovitis in early childhood makes it the first suspected pathology in a limping child. Trauma, which has long been regarded as a causative factor for transient synovitis, may be underestimated in a non-cooperative toddler.

After excluding most serious conditions, such as septic arthritis, a speculative diagnosis of transient synovitis can be made, and this can easily mask a subtle musculoskeletal injury.

**Case presentations:**

We report the cases of three Caucasian patients (two boys, aged 20-months- and three-years-old, and one girl, aged two-years-old), with tibial torus and toddler's fractures which were late-diagnosed due to an initial misdiagnosis of transient synovitis of the hip.

**Conclusion:**

In a non-cooperative child musculoskeletal trauma can be mistaken as a simple causative factor for transient synovitis of the hip and this can easily prevent further investigation for a possible subtle musculoskeletal injury of the lower extremities.

Our experience with the presented cases suggests the need to be more vigilant in the differential diagnosis of transient synovitis in young children.

## Introduction

Toddler's fracture is a subtle, non-displaced fracture of the tibia in children, aged between nine-months-old to three-years-old. The child presents with an acute onset of limp or refusal to bear weight on the leg. Toddlers may be unable to localize pain or give a history. They are also usually uncooperative during the physical exam. Clinical signs of a toddler's fracture can be subtle with non-specific physical findings of local injury.

Transient synovitis (TS) of the hip is one of the most common causes of hip pain and limping during early childhood [1-4]. This benign condition is a clinical diagnosis, which is confirmed by excluding potentially more severe disorders, such as septic arthritis, osteomyelitis, slipped femoral epiphysis and Perthes' disease. Septic arthritis is the first, and occasionally the main condition that most clinicians would like to exclude, due to its devastating course [5,6]. However trauma, which has commonly been mentioned as a causative factor, has probably been underestimated in the differential diagnosis of this frequent entity [3,4,7-11].

We describe three cases of tibial torus and toddler's fractures. The initial misdiagnosis of TS of the hip delayed the true diagnosis.

## Case presentations

### Case #1

**Figure 1:**
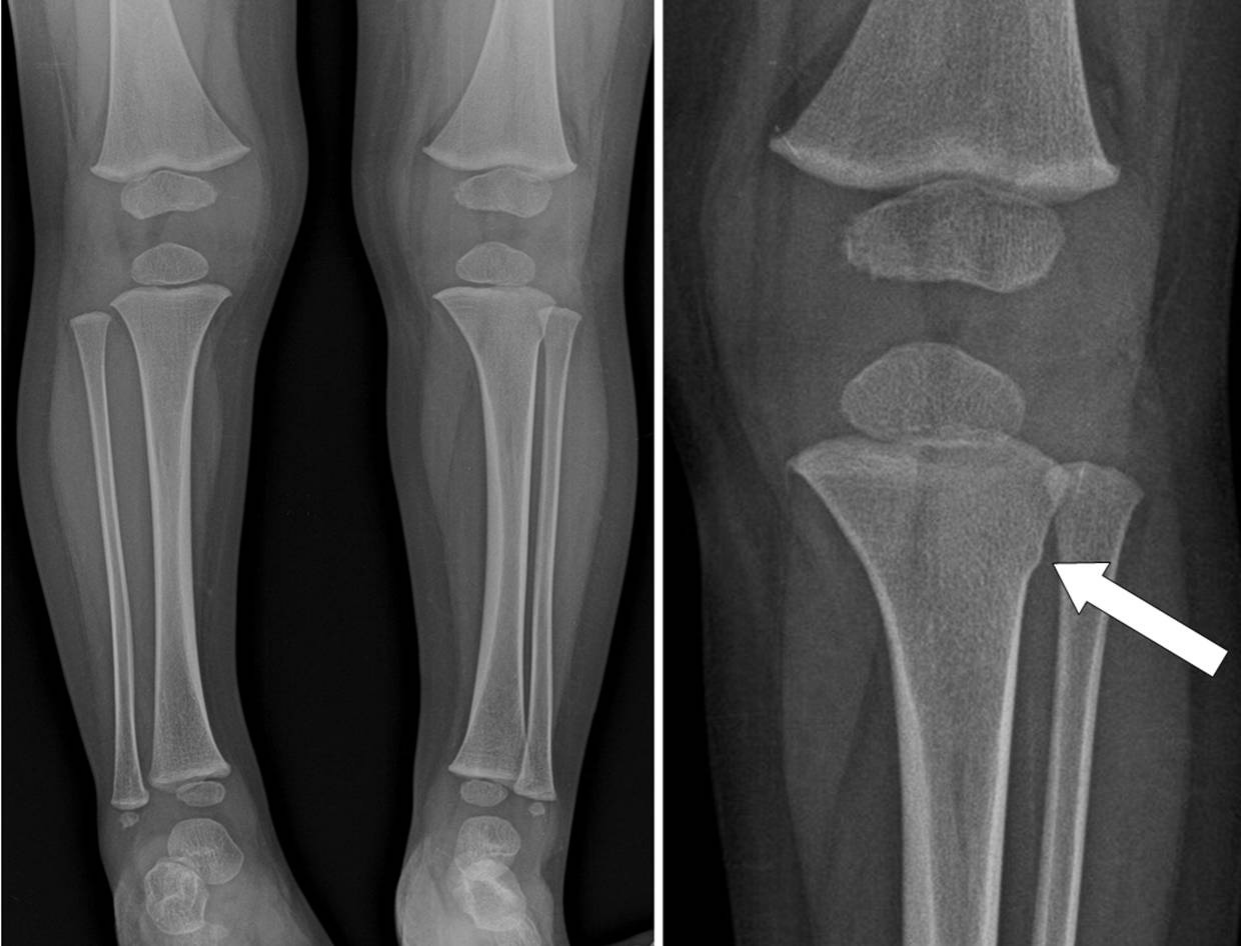
**Whole lower extremity radiograph showed a metaphyseal torus fracture of the left tibia (*arrow*)**.

A 20-month-old Caucasian boy presented with acute left sided lower extremity pain and limping. He was not cooperative and his parents did not mention a history of trauma. He held his hip in flexion and external rotation. No swelling and no signs of inflammation were observed. On physical examination, he had no particularly tender zone, but a generalized referred pain to the entire lower extremity with passive rotations of the left hip. A low-grade fever of 37.2°C was present and his erythrocyte sedimentation rate (ESR) was 12 mm/hour. The anteroposterior and frog leg radiographs of the pelvis were normal, and a probability of TS was suspected. Anti-inflammatory therapy with ibuprofen suspension (100 mg orally twice a day) was administered and the child was discharged to return later for a follow up. Two days after his initial presentation, the child returned with the same symptoms and no relief. On re-examination there was moderate swelling and local tenderness at the lateral aspect of the knee. A whole lower extremity radiograph showed a metaphyseal torus fracture of the left tibia (Figure 1). The boy was treated with a long leg plaster cast for four weeks. Retrospective questioning of the parents revealed a history of an unknown period of care under the supervision of his nursemaid.

### Case #2

**Figure 2:**
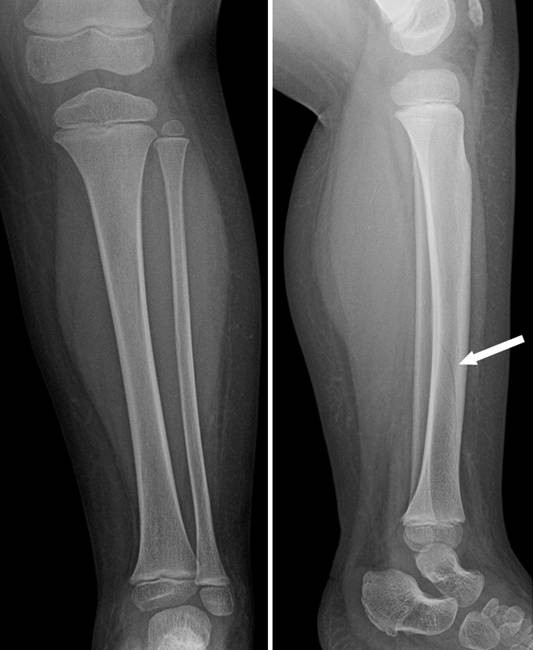
**Whole-leg radiograph of the lower extremity revealed a subtle non-displaced oblique tibia fracture which was only seen on the lateral view (*arrow*)**.

A three-year-old Caucasian boy presented to a local pediatric polyclinic with acute hip pain and difficulty in bearing weight on his left leg. The child had no fever (36.5°C) and his CRP was negative. Radiographic examination of his hip did not reveal any pathology and a diagnosis of TS was made. Bed rest and anti-inflammatory therapy with acetaminophen (120 mg orally, twice a day) was started. He did not improve after six days of treatment, and he was referred to our institution with a suspected diagnosis of an early-onset Perthes disease. On his re-examination, aside from the painful hip rotations, there was local tenderness and swelling in the left leg and passive flexion and extension of the ankle was painful. An entire-leg radiograph of the lower extremity revealed a subtle non-displaced oblique fracture of the left tibia (Figure 2). After the application of a long leg cast, his symptoms subsided dramatically. The fracture healed at the end of the fifth week. Retrospective questioning of the parents confirmed that the patient had a history of losing his footing when running with the dog.

### Case #3

A two-year-old Caucasian girl presented to our emergency room with acute right-sided lower extremity pain and limping. She had stumbled the same morning she was admitted, and her parents noticed her limping late in the afternoon. She was initially evaluated by the emergency room physician and then consulted by a pediatrician in attendance. She held her hip in flexion and no local tenderness was observed. On physical examination, she had a generalized pain which referred to the entire lower extremity and abduction and internal rotation of the hip was limited. She had a slight fever of 37.4°C, her CRP was negative (1.6 mg/L) and her ESR was 8 mm/hour. With the initial diagnosis of TS, she was given an anti-inflammatory (ibuprofen, 100 mg orally, three times a day) and bed rest was advised.

**Figure 3:**
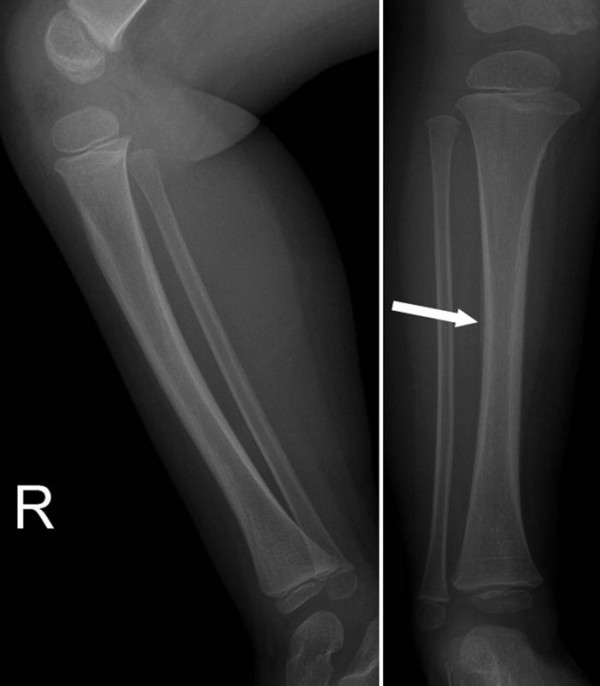
**Lower extremity radiograph showed periosteal new bone formation suggesting healing of a toddler's fracture**.

She had a slight improvement of her symptoms in the first week. Two weeks after her initial visit, she was evaluated in our orthopedic outpatient clinic because of her persisting symptoms. There was local tenderness and swelling on her right leg and passive flexion and extension of the ankle was painful. A whole lower extremity radiograph showed peri-osteal new bone formation suggesting the healing of a toddler's fracture (Figure 3). The patient healed uneventfully after two additional weeks of immobilization and she had no complaint during her follow-up examination three months after her initial admission.

## Discussion

Toddler's fracture was described by Dunbar in 1964 as a subtle, non-displaced fracture of the tibia in children, nine months to three years of age [12]. The child presents with an acute onset of limp or refusal to bear weight on the leg. Toddlers are unsteady and they may fall with a twist, or they may have gotten their foot caught and fallen. The fall is generally unwitnessed by the parents who will be unsure of an injury. Clinical signs of a toddler's fracture can be subtle with non-specific physical findings of local injury. Radiologic signs can also be subtle, as in the presented cases. The fracture may only be seen on the oblique views.

TS of the hip, is an inflammation and swelling of the tissues around the hip joint. It is accepted as the most common cause of sudden hip pain in children. The diagnosis of TS is inevitably speculative and retrospective. The similarities between TS and other more serious diseases makes the diagnosis difficult. The differential diagnosis includes, but is not limited to, the conditions listed in Table 1.

**Table 1:** Differential diagnosis of acute hip pain and limp

• Septic arthritis

• Perthes disease

• Juvenile rheumatoid arthritis

• Discitis

• Psoas abscess

• Stress fracture

• Overuse syndrome

• Rheumatic fever

• Proximal femoral osteomyelitis

• Kawasaki syndrome

• Gaucher disease

• Tumor (Ewing, osteoid osteoma, osteogenic sarcoma, acute lymphocytic leukemia)

• Serum sickness

• Slipped capital femoral epiphysis

• Tuberculosis

**Figure 4:**
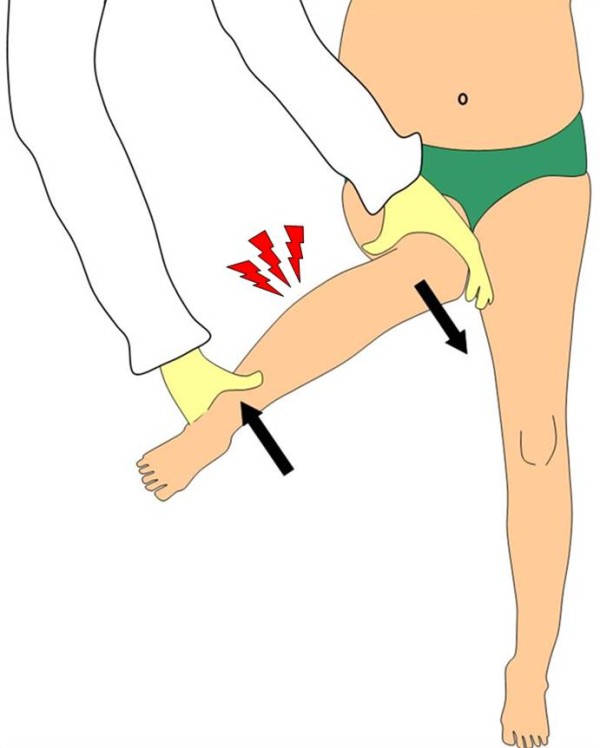
**During the examination of the hip rotations the shear forces acting on the leg (*arrows*) can elicit pain in an injured tibia and this can be mistaken as a hip tenderness**.

Difficulty in bearing weight on a leg, characteristic of an acute onset of a limp, suggests the diagnosis of TS [2,3,8,9,13-15] While limitation of internal rotation is the most common finding, referred pain in the knee can occasionally be the predominant complaint [4]. In the reported cases, passive rotations of the hip joint, tested with 90 degrees of hip and knee flexion, were painful. The reactions of the children were probably due to the leg pain which was obviously triggered by this maneuver (Figure 4).

The difficulties in taking history and evaluation in a young child, the natural association with trauma, and referred pain are all important factors complicating the differential diagnosis of TS in early childhood. The presented young children with toddler's fractures were unable to localize pain or give a history. They were uncooperative during the physical examination.

Trauma has been commonly mentioned as a causative factor for TS [3,4,7-11]. It has been reported to have occurred in, as high as, 17% to 30% of the patients [4]. Local contusion to the hip is thought to set up a self limiting chemical synovitis which resolves as the hematoma is reabsorbed. Trauma history can be considered as a natural preceding condition in TS. This can prevent a thorough investigation for a probable subtle musculoskeletal injury.

While it is difficult to assess the accuracy of published reports on TS, which is obviously an excluding one, the high rates (up to 30%) of trauma history can be due to several missed diagnoses of musculoskeletal injuries.

Finally, we should also mention the probability of the co-existence of TS in the reported cases. While our patients had limited and painful hip rotation at their initial evaluation, it is not clear if this was due to leg pain or an accompanying TS in the hip. Steady relief was observed in their symptoms after immobilization of the leg.

## Conclusion

In non-cooperative young children, musculoskeletal trauma can be mistaken as a simple causative factor for TS of the hip which can easily preclude further investigation for a possible subtle musculoskeletal injury of the lower extremities.

Our experience with the presented cases suggests the need to be more vigilant in the differential diagnosis of TS in early childhood. We believe that a detailed history should be taken from the parents and that a musculoskeletal injury should always be considered, even with a minor trauma history.

## Consent

Written informed consent was obtained from all three patient's parents for publication of these case reports and any accompanying images. A copy of the written consent is available for review by the Editor-in-Chief of this journal.

## Competing interests

The authors declare that they have no competing interests.

## Authors' contributions

All authors read and approved the final manuscript. AS wrote the abstract, introduction, and discussion sections. SU wrote the case report section. BA reviewed the literature, prepared the figures and participated in writing the case reports. MD revised the manuscript and gave final approval of the version to be published.
